# Mars: simplifying bioinformatics workflows through a containerized approach to tool integration and management

**DOI:** 10.1093/bioadv/vbaf074

**Published:** 2025-04-04

**Authors:** Fathima Nuzla Ismail, Shanika Amarasoma

**Affiliations:** Department of Mathematics, State University of New York at Buffalo, Buffalo, NY 14260, United States; Independent Researcher, AI & Advanced Analytics, Colombo 01100, Sri Lanka

## Abstract

**Summary:**

Bioinformatics is a rapidly evolving field with numerous specialized tools developed for essential genomic analysis tasks, such as read simulation, mapping, and variant calling. However, managing these tools presents significant challenges due to varied dependencies, execution steps, and output formats, complicating the installation and configuration processes. To address these issues, we introduce “Mars” a bioinformatics solution encapsulated within a singularity container that preloads a comprehensive suite of widely used genomic tools. Mars not only simplifies the installation of these tools but also automates critical workflow functions, including sequence sample preparation, read simulation, read mapping, variant calling, and result comparison. By streamlining the execution of these workflows, Mars enables users to easily manage input-output formats and compare results across different tools, thereby enhancing reproducibility and efficiency. Furthermore, by providing a cohesive environment that integrates tool management with a flexible workflow interface, Mars empowers researchers to focus on their analyses rather than the complexities of tool configuration. This integrated solution facilitates the testing of various combinations of tools and algorithms, enabling users to evaluate performance based on different metrics and identify the optimal tools for their specific genomic analysis needs. Through Mars, we aim to enhance the accessibility and usability of bioinformatics tools, ultimately advancing research in genomic analysis.

**Availability and implementation:**

Mars is freely available at https://github.com/GenomicAI/mars. It is implemented within a Singularity container environment and supports modular extension for additional genomic tools and custom workflows.

## 1 Introduction

In genomics research, the variety and complexity of tools used to process, analyze, and interpret sequencing data have expanded significantly. Although tools like HTSlib, SAMtools, and BCFtools are widely adopted, the challenges of managing dependencies, ensuring compatibility across different versions, and maintaining efficient workflow delay reproducibility and scalability. A solution that consolidates commonly used tools into a single portable environment could significantly reduce the overhead associated with bioinformatic pipelines. Singularity containers offer such a solution, providing a self-contained an ecosystem that simplifies tool management and ensures version consistency across various computing environments (Kurtzer *et al.* 2017). This review discusses the integration of key genomic tools into a singularity container, which we have developed to standardize and streamline genomic workflows. The tools selected for integration are based on their popularity and utility in whole genome sequencing (WGS) analysis, variant call, and genome assembly. Furthermore, we propose a novel tool named “Mars” to manage and coordinate these utilities, enhancing the automation and usability of the platform.

### 1.1 Tool integration and compatibility

The tools integrated into the singularity container span several critical functionalities to genomic research, such as data compression and indexing (HTSlib, Tabix, bgzip), alignment (BWA, Bowtie2), variant calling (FreeBayes, GATK, Delly) and genome assembly (wfmash, seqwish, vg). These tools have been tested for compatibility within the container and validated to ensure smooth operation.

1. HTSlib, Tabix, and bgzip: These tools form the backbone of many bioinformatics pipelines by enabling efficient compression and indexing of sequence data. HTSlib has been rigorously tested to work seamlessly with the rest of the integrated tools (Bonfield *et al.* 2021).
2. SAMtools, BCFtools, and Bedtools: SAMtools and BCFtools are essential for manipulating SAM/BAM/CRAM files in sequence alignment and variant calling workflows (Danecek *et al.* 2021). Bedtools complements these by providing a suite of utilities for intersecting, merging, and manipulating BED files, which are crucial for analyzing genomic features, such as peaks in ChIP-seq data or genomic intervals in gene expression studies (Quinlan and Hall 2010). Together, these tools streamline genomic data manipulation tasks and ensure a comprehensive set of functions for sequence data analysis.
3. Alignment Tools (BWA, Bowtie2, Minimap2, Diamond): Aligners such as BWA (Li and Durbin 2009) and Bowtie2 (Henriksen *et al.* 2023) are widely used in next-generation sequencing (NGS) for mapping reads to reference genomes, while Minimap2 (Li 2021) provides fast and efficient alignment for both short and long reads, making it essential for hybrid sequencing workflows. Diamond, an accelerated BLAST-compatible aligner, enables high-speed alignment of protein and nucleotide sequences, facilitating rapid comparison and annotation in metagenomic and transcriptomic studies (Buchfink *et al.* 2021).
4. Variant Callers (FreeBayes, GATK, BCFtools): FreeBayes (Garrison and Marth 2012) and GATK (Poplin *et al.* 2018) are prominent tools in variant calling, each using different algorithms for identifying single nucleotide polymorphisms (SNPs) and indels. BCFtools is widely used for processing and manipulating variant calls, making it invaluable for variant-calling workflows (Danecek *et al.* 2021).
5. Genome Graph Tools (vg, seqwish, odgi, gfaffix): Tools such as vg (Hickey *et al.* 2020) and seqwish (Garrison and Guarracino 2023) provide graph-based approaches to represent and analyze pangenomes, capturing structural variations missed by linear reference-based methods. The inclusion of odgi (Guarracino *et al.* 2022) and gfaffix (Doerr *et al.* 2022) enables efficient manipulation of variation graphs, enhancing the versatility of the container for complex genome analyses.
6. Read Simulators (wgsim, ngsngs, pbsim, badread): Simulating sequence data is critical for benchmarking tools and validating workflows. wgsim (Danecek *et al.* 2021), ngsngs (Henriksen *et al.* 2023), pbsim (Ono *et al.* 2022), and badread (Wick 2019) are included to enable the generation of synthetic read data, mimicking real-world sequencing errors and patterns.
7. Assembly and Structural Variation Tools (Delly, wfmash, smoothxg): Delly (Rausch *et al.* 2012) specializes in detecting structural variants such as deletions, duplications, translocations, and inversions, making it an essential tool for understanding large-scale genomic changes. wfmash (Marco-Sola *et al.* 2023) provides rapid alignment for large-scale genome comparisons, and smoothxg (Garrison *et al.* 2024) helps refine assemblies by leveraging variation graphs for more accurate structural variation analysis.
8. Workflow Management (Nextflow): Nextflow is a robust workflow management tool widely used in bioinformatics for creating scalable and reproducible data analysis pipelines (Di Tommaso *et al.* 2017). Its flexibility allows researchers to write workflows in a domain-specific language that can handle complex bioinformatics tasks, such as variant calling, sequence alignment, and genome assembly.

### 1.2 Challenges in tool integration

For bioinformaticians and similar professionals, installing a comprehensive suite of bioinformatics tools individually on a server presents significant challenges. This process requires expertise in software compatibility, dependency management, and command-line configuration across different environments. Here are some key challenges:

1. Dependency Conflicts: Many bioinformatics tools rely on specific versions of underlying libraries (e.g. HTSlib for SAMtools, BCFtools, and Tabix). Installing tools that rely on different versions of these libraries can lead to conflicts, requiring careful version control and frequent adjustments to avoid compatibility issues.
2. Version Management: Bioinformatics tools are frequently updated, and maintaining compatibility with specific versions are crucial for reproducibility in research. Managing version updates manually for each tool, as seen with tools like FreeBayes, GATK, and SAMtools, can be error-prone and time-consuming, especially on shared servers with limited administrative control.
3. System-Specific Constraints: Tools written in different programming languages (e.g. C, Python, Java) may have dependencies specific to those languages, requiring additional installations like JDK for GATK or Python packages for VG. Ensuring that these dependencies are correctly installed and managed for each tool is complex and can be hindered by OS restrictions, library paths, and user permissions.
4. Resource and Performance Optimization: Certain tools, particularly aligners like BWA and Bowtie2, are resource-intensive, and setting up each tool to utilize server resources efficiently is difficult when handling them independently. Configuring optimal runtime parameters requires careful planning and testing, especially in high-performance computing environments.
5. Configuration Consistency: Ensuring that tools such as Delly, Diamond, and Nextflow are consistently configured across multiple projects on the same server is challenging. Differences in configuration files, environment variables, and paths can lead to subtle but impactful inconsistencies in results, requiring continuous monitoring.
6. Scalability and Reproducibility: Running these tools together in workflows adds complexity when each tool is installed separately. A containerized environment can standardize tool versions and configurations, significantly enhancing reproducibility and facilitating large-scale, reproducible analyses that would otherwise require extensive scripting and configuration efforts.

Using a singular container solution, such as Singularity, simplifies these challenges by packaging all tools with their dependencies into one environment, enhancing reproducibility and consistency while minimizing system conflicts. This approach provides bioinformaticians with a controlled environment that is portable across systems, more efficient to deploy, and maintains the reproducibility necessary for bioinformatics research.

### 1.3 The role of “Mars” as a managing tool

The complexity of genomic pipelines necessitates a management layer to automate tool execution and handle data flow between different tools. We propose “Mars” as a unifying command-line interface (CLI) that orchestrates the execution of integrated tools within the container. “Mars” would simplify the user experience by automating tasks such as read alignment, variant calling, and graph assembly. This abstraction layer reduces the need for manual intervention, enabling users to focus on interpreting results rather than managing tool interactions.

**Figure 1.**
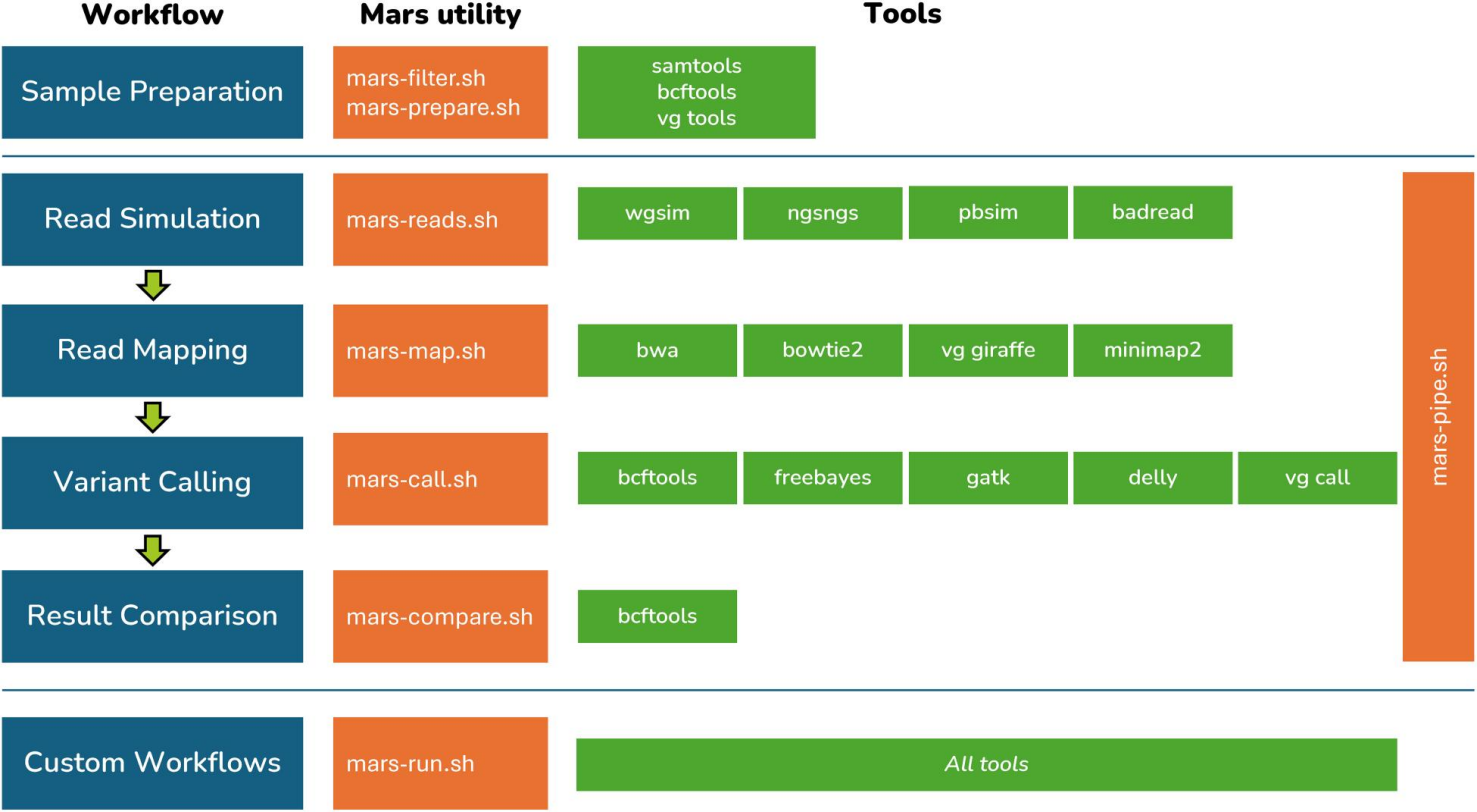
The structure of “Mars.”

The integration of commonly used genomic tools into a Singularity container addresses several of the key challenges in bioinformatics, such as dependency management, version control, and workflow reproducibility. By consolidating these tools into a single platform, we have created a portable, scalable solution for genomic analyses. Additionally, the development of a management tool such as “Mars” will further streamline the process, offering an intuitive interface for complex multistep workflows. This approach enhances the reproducibility of genomic studies and ensures that cutting-edge tools remain accessible to the research community, irrespective of their computational infrastructure (Fig. 1).

“Mars” comes with the following 8 main utilities which will be discussed in the section in detail.

1. Mars run (mars-run.sh): Run any within the Singularity container
2. Mars reads (mars-reads.sh): Simulate reads using different tools available
3. Mars map (mars-map.sh): Align reads to a reference sequence
4. Mars call (mars-call.sh): Call variants using different methods
5. Mars compare (mars-compare.sh): Compare the output with ground truth data
6. Mars pipe (mars-pipe.sh): Master script for running pipeline
7. Mars filter (mars-filter.sh): Filter and index a chromosome from a fasta file
8. Mars prepare (mars-prepare.sh): Create ground truth data files

## 2 Methods

The Mars singularity container incorporates a comprehensive suite of bioinformatics tools essential for genomic data analysis, as described in Table 1. Each tool is integral to various stages of data processing and analysis, from sequence alignment to variant calling. A brief description of these tools follows.

**Table 1.** Tools available in Mars singularity container.

No.	Tool	Version	Execute
1	htslib, tabix, bgzip	1.21	./mars-run.sh tabix -h, ./mars-run.sh bgzip -h
2	SAMtools	1.21	./mars-run.sh samtools version
3	BCFtools	1.21	./mars-run.sh bcftools version
4	BEDtools	v2.31.1	./mars-run.sh bedtools—version
5	wgsim	1.21	./mars-run.sh wgsim -h
6	ngsngs	v0.9.2.2	./mars-run.sh ngsngs -v
7	pbsim	v3.0.4	./mars-run.sh pbsim
8	badread	v0.4.1	./mars-run.sh badread—version
9	bwa	0.7.18-r1243-dirty	./mars-run.sh bwa
10	bowtie2	2.5.4	./mars-run.sh bowtie2—version
11	freebayes	v1.3.6	./mars-run.sh freebayes -h
12	minimap2	2.28-r1209	./mars-run.sh minimap2—version
13	wfmash	v0.21.0	./mars-run.sh wfmash -h
14	seqwish	v0.7.11–0-g0eb6468	./mars-run.sh seqwish -v
15	smoothxg	v0.8.0–0-g66b17ae	./mars-run.sh smoothxg -v
16	gfaffix	0.1.5b	./mars-run.sh gfaffix -V
17	odgi	v0.9.0–3-g237fc1b0	./mars-run.sh odgi
18	vg	v1.60.0	./mars-run.sh vg help
19	gatk	4.6.0.0	./mars-run.sh gatk—list
20	delly	1.2.9	./mars-run.sh delly
21	diamond	v2.1.9.163	./mars-run.sh diamond help
22	nextflow	24.09.2-edge	./mars-run.sh nextflow info

1. **htslib, tabix, bgzip—**A set of C library utilities for handling high-throughput sequencing data formats like BAM and VCF. tabix indexes tab-delimited files, and bgzip compresses and indexes them for efficient querying.
2. **SAMtools—**A toolkit for manipulating sequence alignment data in SAM, BAM, and CRAM formats, used for sorting, indexing, and converting files, as well as variant calling.
3. **BCFtools—**Utilities for manipulating and analyzing Variant Call Format (VCF) and Binary Call Format (BCF) files, commonly used in variant calling and genotyping.
4. **BEDtools—**A suite of tools for genome arithmetic, useful for finding intersections, unions, and other set operations on genomic intervals in BED format.
5. **wgsim—**A tool for simulating short reads from reference genomes to evaluate variant calling pipelines and other genomic analyses.
6. **ngsngs—**A next-generation sequencing simulator that generates realistic sequence data, often used in testing sequencing software and workflows.
7. **bwa—**The Burrow-Wheeler Aligner, a tool for fast and accurate short-read alignment to a reference genome, particularly suited for high-throughput sequencing.
8. **bowtie2** - A fast aligner for short-read data, sensitive to both local and global alignments and capable of gapped alignment.
9. **freebayes—**A Bayesian-based tool for detecting genetic variations and haplotypes in short-read sequencing data, capable of population-based calling.
10. **vg—**A suite for working with genome variation graphs, allowing for complex representations of genetic variation beyond linear genomes.
11. **GATK (Genome Analysis Toolkit)—**A powerful toolkit for variant discovery and genotyping, providing tools for data preprocessing, variant calling, and quality control.
12. **pbsim—**A simulator for generating long reads similar to those produced by PacBio and Oxford Nanopore Technologies, useful for testing long-read analysis workflows.
13. **minimap2—**A versatile aligner for pairwise alignment of genomic and transcriptomic sequences, supporting spliced alignments for RNA-seq data.
14. **wfmash—**An aligner for base-accurate DNA sequences using the Wavefront Alignment and MashMap algorithm for efficient sequence comparison.
15. **seqwish—**A tool for generating variation graphs from read alignments to a reference genome, enabling complex representations of sequence variation.
16. **smoothxg—**A tool for simplifying variation graphs by linearizing and blocking sequences, which aids in producing readable and efficient genome representations.
17. **gfaffix—**A tool for collapsing redundant sequences in genome graphs by identifying shared affixes, resulting in a more compact genome graph structure.
18. **odgi—**A toolkit for optimizing, analyzing, and visualizing dynamic genome graphs, particularly for handling complex pangenomic data.
19. **badread—**A simulator that generates synthetic long reads, introducing realistic sequencing errors, which is useful for testing error-tolerant algorithms.
20. **delly—**A tool for structural variant detection, integrating information from paired-end and split-read sequencing data to identify large structural variants.
21. **diamond—**A high-speed sequence aligner for protein and translated DNA searches, particularly useful in metagenomics and functional annotation.
22. **nextflow—**A workflow management system designed for reproducibility and scalability in bioinformatics, enabling complex and multi-step pipelines to be run locally or in the cloud.

### 2.1 Mars run (mars-run.sh)

The mars-run.sh script is a wrapper utility for executing bioinformatics tools within the Mars Singularity container, as described in Table 1, ensuring all commands run in a consistent, controlled environment. It simplifies command execution by allowing users to call specific tools directly without manually handling dependencies or setting up the environment. Users can use mars-run.sh with various supported commands, enabling them to focus on their analyses rather than container management. Mars Singularity container makes it easy to use a broad range of bioinformatics tools pre-packaged within mars by simply prefixing commands with mars-run.sh.

### 2.2 Mars reads (mars-reads.sh)

The mars-reads.sh script is designed to facilitate the simulation and processing of sequencing reads within the Mars container. It allows users to generate synthetic reads from reference genomes and seamlessly handle steps like read mapping and variant calling. By invoking mars-read.sh, users can execute these tasks in a streamlined manner, ensuring consistency in the workflow. This script simplifies read preparation and processing, making it easy to integrate read simulations and downstream analyses in a single, unified command.

### 2.3 Mars map (mars-map.sh)

The mars-map.sh script in the Mars container is focused on mapping sequencing reads to a reference genome. It manages the alignment process, typically using tools like BWA, Bowtie2 and Minimap2, to positions reads against the reference. This script streamlines the mapping stage, handling necessary parameters and settings ensuring that users can perform read alignment efficiently within the larger workflow. By standardizing the mapping process, mars-map.sh supports reproducible results and is an integral part of the pipeline from read simulation to downstream analyses.

### 2.4 Mars call (mars-call.sh)

The mars-call.sh script in the Mars container is responsible for variant calling, identifying genetic variations such as single nucleotide polymorphisms (SNPs) and structural variants between the sample reads and the reference genome. Using variant-calling tools, this script analyzes the aligned reads to detect variations, generating a comprehensive list of variants in a standardized format. mars-call.sh simplifies the complex variant-calling process, enabling users to systematically pinpoint differences at the genetic level. This script is crucial for interpreting genetic diversity and is a key component in the end-to-end workflow within the Mars pipeline.

### 2.5 Mars compare (mars-compare.sh)

The mars-compare.sh script in the Mars container is designed for evaluating the accuracy of SNPs (single nucleotide polymorphisms) and INDELs (insertions and deletions) identified during variant calling. It compares the detected variants against a known set of true variants to assess the performance of the variant-calling process. This script generates performance metrics such as Sensitivity (true positive rate), Specificity (true negative rate), and F1 Score (harmonic mean of precision and recall), providing a quantitative evaluation of the accuracy and reliability of the variant calls. These metrics are output as matrices, enabling users to interpret the quality of variant detection and optimize settings as needed for improved precision in genetic analyses.

### 2.6 Mars pipe (mars-pipe.sh)

The mars-pipe.sh script serves as a master script within the Mars container, orchestrating the entire bioinformatics pipeline from start to finish. This script sequentially executes each module—sample preparation, read simulation, read mapping, variant calling, and result comparison—ensuring a streamlined, automated workflow. By coordinating these steps, mars-pipe.sh enables users to perform comprehensive genomic analysis in a single run, facilitating the identification and evaluation of variants with minimal manual intervention. The script is ideal for handling large datasets, ensuring reproducibility, and enhancing efficiency in bioinformatics research.

### 2.7 Additional scripts of Mars

The mars-filter.sh, mars-prepare.sh, and mars-graph.sh scripts are additional tools provided within the Mars workflow to support data preparation. These scripts enhance data handling flexibility and are designed to streamline specific steps in variant analysis workflows. Examples of their usage are available in the Git repository, making it easy for users to incorporate them into their analysis processes.

### 2.8 Example workflows

**Figure 2.**
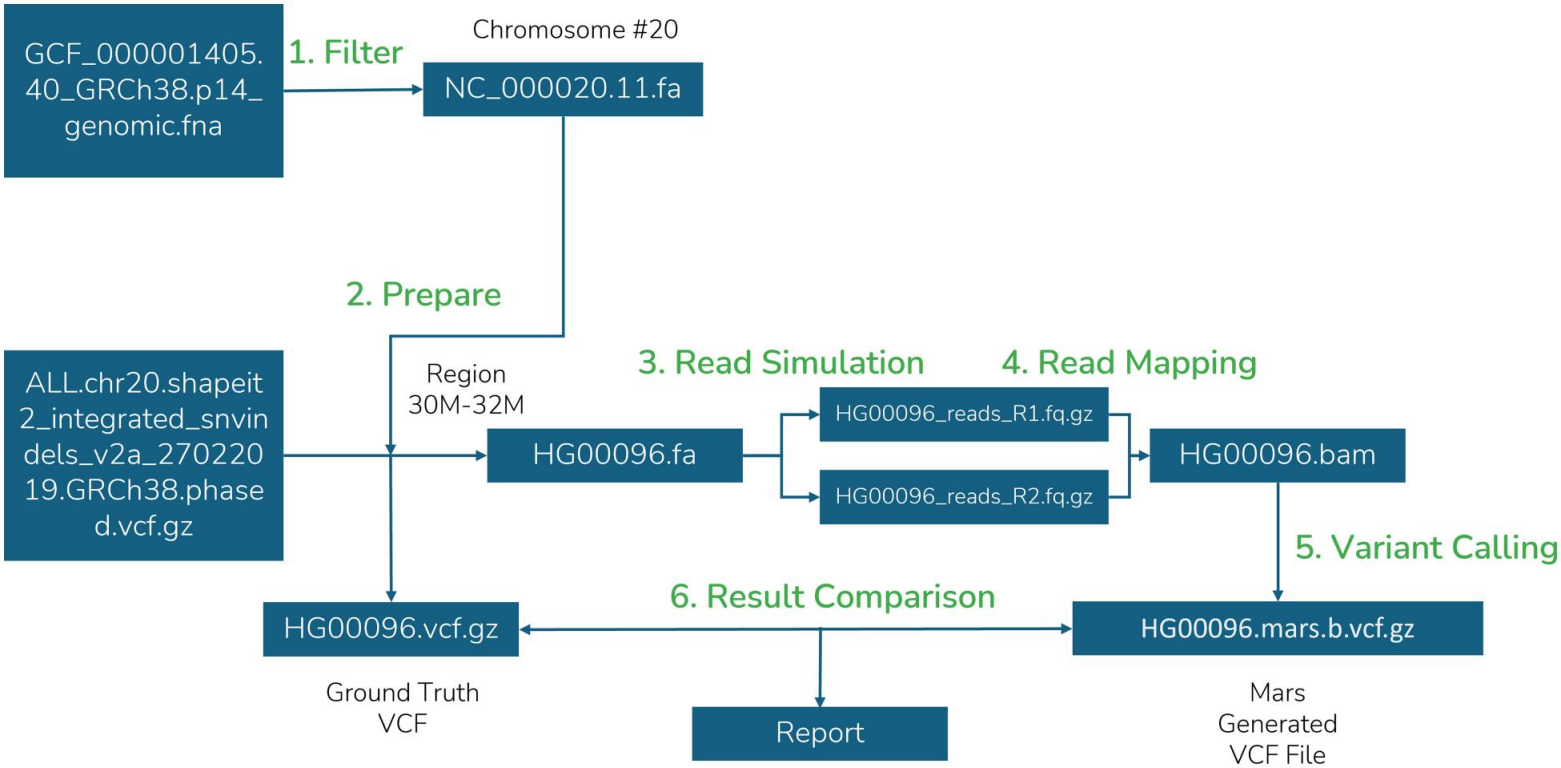
Linear mapping approach example workflow.

**Figure 3.**
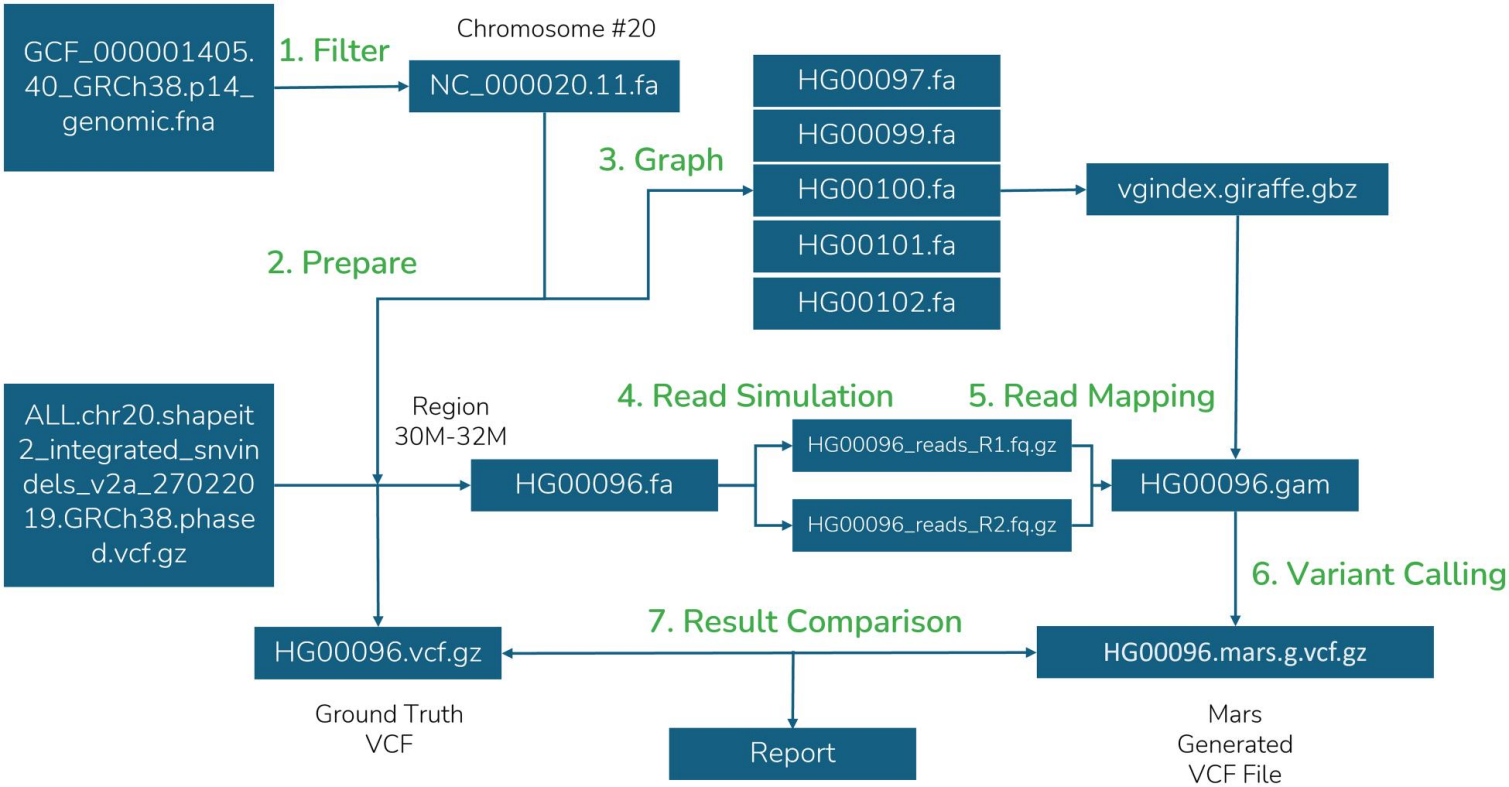
Graph mapping approach example workflow.

The example workflow, “Example Using Genome Assembly GRCh38.p14 Chromosome 20” (https://github.com/GenomicAI/mars? tab=readme-ov-file#example-using-genome-assembly-grch38p14-chromosome-20), demonstrates a comprehensive run-through of the Mars pipeline using human chromosome 20 from the GRCh38.p14 genome assembly. This example guides users through each step of the workflow, as shown in Figs 2 and 3, from sequence simulation to variant comparison, providing a practical demonstration of how to use the Mars tool for bioinformatics analyzes. It includes commands to prepare sequence data, simulate reads, map these reads, perform variant calling, and evaluating results by comparing detected SNPs and INDELs. The example serves as a tutorial and includes sample input files and expected outputs, making it easy for users to understand the entire pipeline process and validate its performance on real genomic data.

The example Nextflow workflow (https://github.com/GenomicAI/mars? tab=readme-ov-file#4-executing-workflow-with-nextflow) included in the Mars repository complements the mars-pipe.sh script by enabling users to handle more complex tasks in a structured manner. This workflow is designed to allow for the integration of various bioinformatics tools and processes, making it easier to explore different features of Nextflow within the Singularity container. Users can experiment with the provided tools and workflows, refining their approach based on the results. This experimentation can serve as a foundation for scaling up to larger, more comprehensive solutions in future analyses.

The structural variant (SV) detection example (https://github.com/GenomicAI/mars? tab=readme-ov-file#structural-variant-sv-detection-workflow) provided in the Mars repository showcases the capability of the tool to identify and analyze structural variants within genomic data. This example illustrates the workflow for detecting SVs using simulated data and integrates various scripts within the Mars suite to preprocess the data, map the reads, and call variants. By following this example, users can gain insights into the process of SV detection, leveraging the combined functionality of the scripts to facilitate their own analyses of structural variants in more complex datasets.

### 2.9 Building the singularity container

The creation of this Singularity container (https://github.com/GenomicAI/mars? tab=readme-ov-file#how-to-build-the-singulairty-container) represents a significant effort by researchers to integrate a wide array of bioinformatics tools into a single, streamlined package. By successfully consolidating multiple applications, the final SIF file size remains under 2GB, a remarkable feat considering that many singularity images for individual tools often exceed this size. This minimal footprint enhances efficiency and simplifies deployment for bioinformaticians, who often face challenges in creating such comprehensive environments. The definition file for this container is available in the Git repository, serving as a practical example for others in the field and encouraging them to build their containers and customize them with additional tools as needed.

## 3 Results and Discussion

### 3.1 Rationale for containerization, integration, and dependency management in Mars

Bioinformatics analyses inherently rely upon diverse specialized software tools for critical genomic tasks such as read simulation, mapping, and variant calling. Typically, individually installing, configuring, and maintaining these tools introduces considerable complexity, often resulting in software incompatibilities, version conflicts, unreproducible software states, and difficulty in portability across different computational environments. Such complexities significantly reduce research productivity, hamper reproducibility, and create obstacles for researchers of varying bioinformatics expertise (Alser *et al.* 2024).

Mars addresses these widespread challenges by leveraging containerization technology, specifically through Singularity, to encapsulate all required bioinformatics tools including commonly used software such as BWA, SAMtools, BCFtools, Freebayes, minimap2, GATK, Delly, wgsim, pbsim, badread, vg giraffe, and vg call along with their dependencies into a single, portable, and self-contained image. By encapsulating these tools, Mars ensures a stable, robust, reproducible software environment that can reliably run across diverse computational platforms (compute clusters, desktops, cloud infrastructure) without complicated installations, dependency resolutions, or management challenges.

Unlike traditional approaches involving direct installations or widely used frameworks like Conda, Mars emphasizes highly integrative, rigorously tested combinations of software tools that prevent runtime dependency conflicts, version incompatibilities, and unexpected conflicts. While environments like Conda offer flexibility in choosing software versions, this flexibility frequently introduces complex dependency resolution challenges (Alser *et al.* 2024). Mars explicitly prioritizes stability, reproducibility, and usability over Conda’s granular software-version flexibility, consciously selecting specific combinations of fully validated, harmonized software components. The build definition file (mars.def) transparently details exact software tools and versions, ensuring clarity and reproducibility by openly documenting this information on the Mars GitHub repository. This transparency encourages reproducible research practices, benefiting researchers who need to be assured of documented computational setups.

Mars further integrates specialized workflow utilities specifically, dedicated wrapper scripts (mars-reads.sh, mars-map.sh, mars-call.sh, mars-compare.sh, and mars-pipe.sh) to streamline end-to-end genomic analyses. These integrated utilities enable rapid, straightforward execution of everyday genomic tasks (such as short and long-read simulation, alignment, variant calling, comparative assessment of variant calls, and pipeline execution), significantly reducing the need for extensive bioinformatics experience, manual intervention, custom scripting, or iterative troubleshooting. As a result, Mars considerably lowers technical barriers, enabling researchers at varying skill levels to focus on biological interpretation rather than computational management and troubleshooting complexities.

### 3.2 Performance benchmarking and computational overhead

To rigorously evaluate whether containerization introduced computational overhead, end-to-end comparative benchmarks between Mars workflows and equivalent noncontainerized (direct installation) setups were conducted (fully documented on the Mars GitHub repository). These assessments examined standard variant calling workflows, for instance, utilizing BWA-MEM alignment followed by BCFtools variant calling within containerized and noncontainerized environments (Amarasoma and Ismail 2024). Results demonstrated minimal computational overhead from container usage, with Mars workflows showing runtime performances within approximately 5%.

### 3.3 Scalability evaluation

Mars’ scalability was comprehensively validated using publicly available genomic datasets (e.g. human genome assembly GRCh38 at varying regional extents of 1–2 Mbp, 20–50Mbp, and 100Mbp endpoints), as documented openly on the Mars GitHub repository. Evaluations encompassed a range of everyday bioinformatics tasks, including sequence-read simulation (wgsim, pbsim, badread), alignment (BWA, minimap2, vg giraffe), and variant calling (BCFtools, Freebayes, vg call, GATK, and Delly). Mars maintained stable, consistent, and predictable performance scaling across small, medium, and more extensive genomic sequence analyses, demonstrating robust applicability in diverse real-world bioinformatics analysis scenarios.

### 3.4 Research contribution and significance

We acknowledge that skilled bioinformatics researchers might already possess the expertise to install and configure bioinformatics tools using Conda environments, manage custom scripts, or resolve software incompatibilities. Nevertheless, Mars explicitly addresses critical challenges experienced broadly across the bioinformatics user community, reducing complexity, dramatically enhancing reproducibility, standardizing workflows, and significantly improving usability. By consolidating tools within rigorously pre-configured Singularity containers, Mars eliminates subtle dependency-related discrepancies, removes the need for iterative troubleshooting, reduces complexity from script customizations, and dramatically improves workflow standardization.

Mars also contributes practically valuable innovations through immediate-deployment capability, comparative evaluation utilities (e.g. mars-compare.sh), incorporation of advanced methods (such as graph-based pangenome variant calling workflows via vg giraffe and vg call), and fully transparent end-to-end validation testing documented openly on GitHub. Collectively, these represent meaningful advancements, improving genomic research workflow execution by addressing practical reproducibility concerns and facilitating rigorous comparative bioinformatics tasks typically overlooked or challenging in typical noncontainerized research scenarios.

Mars strategically prioritizes reproducibility, portability, standardized usability, and integrated workflow solutions rather than arbitrary software-version customization. Through openly available comprehensive documentation, fully transparent container definitions (mars.def), thorough benchmarking, scalability evaluations, reproducibility validations, and workflow standardization tools provided via easily accessible Singularity containers and integrative wrapper utilities, Mars clearly establishes compelling rationale as a robust solution tailored toward addressing common bioinformatics community-wide challenges.

## 4 Conclusion

In conclusion, the “Mars” project is a testament to the successful integration of cutting-edge bioinformatics tools into a user-friendly and cohesive framework, all encapsulated within a Singularity container. The research team has made significant progress in bringing together many vital tools, developing a container with a small footprint, and simplifying the deployment and sharing process for bioinformaticians. This user-friendly method serves as a helpful guide for those interested in constructing their containers using additional tools. The process, which includes read simulation, mapping, variant calling, and result comparison scripts, can be customized to simplify complex bioinformatics investigations, thanks to the flexibility and modularity of these scripts. The integration of Nextflow tools further enhances the project’s user-friendliness, allowing users to experiment with various features without the burden of intricate setups, thereby promoting a more intuitive exploration of advanced computational techniques.

With the prospect of adding new tools and integrating generative AI characteristics, the Mars project is poised to unlock endless possibilities for innovation and improved bioinformatics capabilities. This forward-looking approach not only addresses present issues but also establishes a robust foundation for future developments in the field. The Mars project is set to empower scientists to effectively navigate the intricacies of genetic analysis and beyond, sparking excitement and optimism for the future of bioinformatics.

## Supplementary Material

vbaf074_Supplementary_Data
